# Bio-Based Resin Reinforced with Flax Fiber as Thermorheologically Complex Materials

**DOI:** 10.3390/polym8040153

**Published:** 2016-04-19

**Authors:** Ali Amiri, Arvin Yu, Dean Webster, Chad Ulven

**Affiliations:** 1 Department of Mechanical Engineering, North Dakota State University, Fargo, ND 58102, USA; ali.amiri@ndsu.edu; 2 Department of Coatings and Polymeric Materials, North Dakota State University, Fargo, ND 58102, USA; arvin.yu@ndsu.edu (A.Y.); dean.webster@ndsu.edu (D.W.)

**Keywords:** composites, creep, ester synthesis, flax fiber, time-temperature superposition

## Abstract

With the increase in structural applications of bio-based composites, the study of long-term creep behavior of these materials turns into a significant issue. Because of their bond type and structure, natural fibers and thermoset resins exhibit nonlinear viscoelastic behavior. Time-temperature superposition (TTS) provides a useful tool to overcome the challenge of the long time required to perform the tests. The TTS principle assumes that the effect of temperature and time are equivalent when considering the creep behavior, therefore creep tests performed at elevated temperatures may be converted to tests performed at longer times. In this study, flax fiber composites were processed with a novel liquid molding methacrylated epoxidized sucrose soyate (MESS) resin. Frequency scans of flax/MESS composites were obtained at different temperatures and storage modulus and loss modulus were recorded and the application of horizontal and vertical shift factors to these viscoelastic functions were studied. In addition, short-term strain creep at different temperatures was measured and curves were shifted with solely horizontal, and with both horizontal and vertical shift factors. The resulting master curves were compared with a 24-h creep test and two extrapolated creep models. The findings revealed that use of both horizontal and vertical shift factors will result in a smoother master curves for loss modulus and storage modulus, while use of only horizontal shift factors for creep data provides acceptable creep strain master curves. Based on the findings of this study, flax/MESS composites can be considered as thermorheologically complex materials.

## 1. Introduction

### 1.1. Natural Fibers and Bio-Based Resins

**Figure 1:**
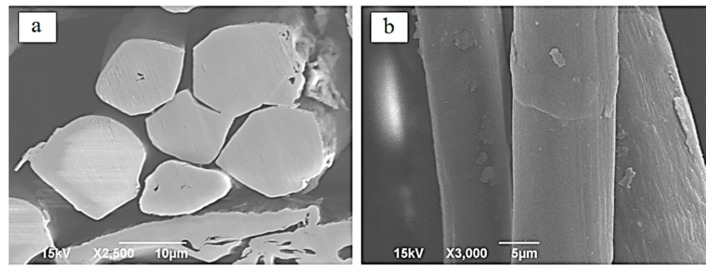
SEM images of (**a**) the cross-section of flax fiber and (**b**) the surface of the fiber used in this study.

Because of environmental impact of natural fibers [1], and having lower density, higher toughness and comparable specific strength and specific stiffness to mineral fibers, such as glass fiber, bio-based composites are considered a preferred alternative to conventional composites [2,3]. When using natural fibers it is important to understand that unlike synthetic or mineral-based fibers, natural fibers exhibit nonlinear elastic behavior. The nonlinearity in elastic behavior is the result of the natural fiber’s structure. The multiple layers (primary and secondary walls) that make up the natural fiber’s structure lead to the viscoelastic behavior. In a single fiber, there are several different layers that make up the fiber’s structure, which are the primary wall, secondary wall, and the center lumen [4,5]. Figure 1 shows a typical cross-section and surface of flax fiber used in this study, obtained by Scanning Electron Microscopy (SEM). Flax fiber is one of the common natural reinforcements used in bio-based reinforced composites [4] and, in recent years, there have been numerous research efforts on flax fiber reinforced composites [6,7,8,9,10,11,12].

In addition, the replacement of the petroleum-based polymers with their bio-based counterparts is a recent innovation in the field of “green composites” [13,14]. Over the last decade, a broad range of chemical routes to utilize natural triglyceride oils to create synthetic-like polymer structure as a basis for coatings, inks, plasticizers, lubricants, and polymers materials has been developed. In this study, a novel high-functional bio-based resin from methacrylated epoxidized sucrose soyate (MESS) was used as a matrix [15]. The synthesis of the MESS resin is discussed in the Materials and Methods.

Bio-based composites can be divided into continuous fiber composite and discontinuous fiber composites types, based on their fiber length, and are further divided into partially or fully bio-based by their degree of biodegradability [16]. Flax/MESS composite under study here can be fit into the continuously reinforced and fully bio-based category.

### 1.2. Long-Term and Thermal Behavior

As there has been an increase in the structural applications of bio-based composites; studying the long-term creep behavior of these materials turns into an important aspect of their development [17]. When studying creep behavior of composite materials, Time-Temperature Superposition (TTS) is a useful tool to overcome the challenges of the long time required to perform the tests [1,17]. The TTS principle assumes that the effect of temperature and time are equivalent when considering the viscoelastic behavior [18]. The creep data obtained at different temperatures are shifted horizontally, relative to a curve at a reference temperature, to superimpose and generate a master curve. The creep master curve provides information regarding behavior of the material under study over a wide range of times [19]. Shift factors are in the form of either Williams-Landel and Ferry (WLF) [18] or Arrhenius [20] Equations, where the shift in temperature is related to the shift in time. In addition, based on Ward [21] and Van Gurp *et al.* [22], the same shift factors should be able to superpose all viscoelastic properties. In addition, the creep test’s temperature range should be below that of material degradation, and there should be no residual curing of the resin occurring during the creep tests. Moreover, for crystalline and semi-crystalline materials, the deformation should be kept in the linear viscoelastic range by applying low strains [1]. To achieve a smooth master curve for semi-crystalline materials, there might also be a need for empirical vertical shift factors [23]. According to Djokovic *et al.* [24], amorphous materials are thermorheologically simple and a horizontal shifting of creep data is enough to achieve a master curve, and crystalline materials are thermorheologically complex and both horizontal and vertical shift factors are needed. It is not very clear how to categorize thermoset resins reinforced with natural fibers, such as flax, into simple or complex materials. This issue is the focus of this paper.

### 1.3. Creep Modeling

Thermoset resins reinforced with flax fiber exhibit non-linear behavior when subjected to loading [11,25]. In addition, the inherent variation present in natural flax fiber [5], as well as polymer matrices, make the design of bio-based composites a complex task. Therefore, empirical models and results are widely used to help designers with their applications [16].

#### 1.3.1. Nutting Power Law

Nutting [26], in 1921, proposed an empirical strain-stress-time model, which showed good agreement with steady state creep for metals:

(1) $$\varepsilon^{c}=k\sigma^{P}t^{n}$$

where $\varepsilon^{c}$ is the creep strain, σ is the applied stress, *k*, *P* and *n* are temperature-dependent material constants. This model has been used by researchers for viscoelastic materials with satisfactory results for short-term creep [20,27,28,29].

#### 1.3.2. Findley Power Law

According to Findley [30], the time-dependent creep compliance, *J(t)*, of a material can be represented by a power function as:

(2) $$J=J_{o}+J\left(t\right)=J_{0}+A\;t^{n}$$

where *J*_0_ is the time-independent or elastic creep compliance, *A* is the time-dependent coefficient, t is the time and *n* is stress-independent coefficient. Creep compliance *J(t)* in flexure is defined as the time-dependent strain per unit stress [31]. Therefore, Equation (2) can be modified as follows to represent the entire strain creep curve of a material, ε, can be expressed as:

(3) $$\varepsilon =\varepsilon_{0}+\varepsilon \left(t\right)$$

where $\varepsilon_{0}$ is the elastic strain, and ε(t) is the time-dependent strain. From the definition of creep compliance and Equation (2):

(4) $$J\left(t\right)=\frac{\varepsilon \left(t\right)}{\sigma_{0}}=At^{n}$$

Therefore, the modified Findley Power Law for strain creep can be presented as follows:

(5) $$\varepsilon =\varepsilon_{0}+A\sigma_{0}t^{n}$$

In previous studies [1,17] it was shown that the time–temperature superposition principle is applicable to natural fiber/thermoset resins, and that the shift factors follow the Arrhenius relation. However, the validity of applicability of the same shift factors to other viscoelastic functions was not studied. The resin used in previous studies was vinyl ester. The mechanical properties of flax/vinyl ester was investigated and compared with the new MESS resin reinforced with flax fiber in another study [5]. The focus of this paper is to further study the thermal behavior of the novel bio-based composites made using the MESS resin. Frequency scans of flax/MESS composites were obtained at different temperatures and storage modulus, loss modulus and tan δ (the ratio of loss to storage modulus) were recorded. Application of horizontal and vertical shift factors to all these viscoelastic functions were studied. In addition, short-term strain creep at different temperatures was measured and curves were shifted both with only horizontal, and with both horizontal and vertical shift factors. The resulting master curves were compared with a 24-h creep test and two creep models that were mentioned previously. The results of the current study are complementary to the existing information for natural fiber/thermoset resin composites and newly developed MESS resin.

## 2. Materials and Methods

Fiber used in this study was Biotex Flax 2 × 2 twill fabric mat with the areal density of 400 g/m^2^ obtained from Composites Evolution, Chesterfield, UK. Methacrylated epoxidized sucrose soyate (MESS) resin was made by the reaction of epoxidized sucrose soyate (ESS) and methacrylic acid. ESS is 100% bio-based (containing no epichlorohydrin) and was synthesized from fully esterified sucrose soyate as reported previously in [15,32,33].

**Figure 2:**
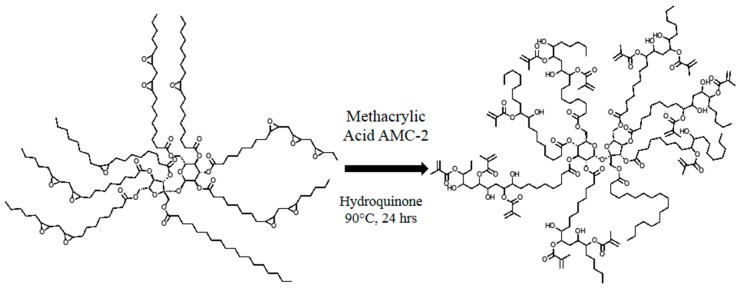
Synthetic route to MESS.

The synthesis of the resin was achieved via ring-opening reaction of ESS with methacrylic acid. The reaction was carried out at 90 °C, using AMC-2 (source) as the catalyst and hydroquinone as the inhibitor. The molar ratio of methacrylic acid (source) to epoxy was 0.8. The procedure is as follows: ESS (1000.00 g), methacrylic acid (291.831 g, acid to epoxy ratio = 0.8), hydroquinone (source) (6.459 g, 0.5% of total weight) and AMC-2 (12.918 g, 1.0% of total weight) were placed into a three-necked, round-bottomed flask equipped with a mechanical stirrer and a thermocouple. The mixture was heated at 90 °C for 24 h. The final resin appeared as a dark green, viscous liquid. No further purification was carried out before incorporation into the composites. The synthesis rout is presented in Figure 2 [32].

The MESS resin was too viscous to be used for thermoset formulations. Therefore, styrene was introduced as a reactive diluent to reduce the viscosity, as well as a co-monomer to increase the rigidity of the resulting thermoset. The resulting resin contained 30% styrene. The resin was mixed with tert-butyl peroxybenzoate 98% (Luperox^®^ P) as a high temperature initiator and cumyl hydroperoxide 45% (Trigonox 239A) as a room temperature initiator. The mixing ratio of Luperox^®^ P, Trigonox 239A were 2 and 3 wt %, respectively. Styrene and Luperox^®^ P were purchased from Sigma-Aldrich Co. located in St. Louis, MI, USA. Cumyl Peroxide, commercially available as Trigonox 239A, was generously provided by AkzoNobel Co., Amsterdam, Netherlands.

### 2.1. Characterization of MESS

Fourier transform infrared spectroscopy (FTIR) was performed on the resin with a Thermo Scientific Nicolet 8700 with a detector type of DTGS KBr under nitrogen purge. Diluted thin films of the samples were applied on a KBr plate and the absorption spectra were taken with 32 scans at a resolution of 4 cm^−1^. Molecular weight of the resin was obtained using a gel permeation chromatography (GPC) system (EcoSEC HLC-8320GPC, Tosoh Bioscience, Tokyo, Japan) with a differential refractometer (DRI) detector. Separations were performed using two TSKgel SuperH3000 6.00 mm ID × 15 cm columns with an eluent flow rate of 0.35 mL·min^−1^. The columns and detectors were thermostated at 40 °C. The eluent used is tetrahydrofuran (THF). Resin samples were prepared at nominally 1 mg·mL^−1^ in an aliquot of the eluent and allowed to dissolve at ambient temperature for several hours and the injection volume was 20 µL for each sample. Calibration test of the resin was conducted using polystyrene standards (Agilent EasiVial PS-H 4 mL, Santa Clara, CA, USA). Proton nuclear magnetic resonance spectroscopy (^1^H NMR) was conducted with a Bruker system, Ascend 400 MHz magnet with an Avance III HD console (Bruker BioSpin Corporation, Billerica, MA, USA), using CDCl_3_ as the solvent. Acid number titration was carried out according to ASTM D664. The viscosity of the resins was measured at 25 °C using an ARES Rheometer (TA Instruments, New Castle, DE, USA) operating from 0.1 to 500 rad/s with 0.1% strain.

### 2.2. Composite Preparation

**Figure 3:**
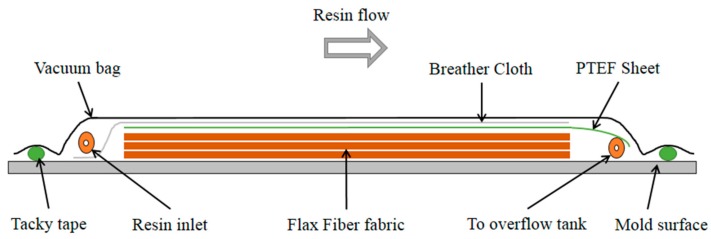
Schematic of the Vacuum Assisted Resin Transfer Molding (VARTM) set-up used to manufacture composite sample.

To manufacture the composite plate, Vacuum Assisted Resin Transfer Molding (VARTM) method was used. The mold surface was waxed with mold release agent and six layers of flax fiber mat were stacked up on each other. One layer of polytetrafluoroethylene (PTFE) and one layer of breather cloth were placed on top of all materials. Finally, a layer of vacuum bagging film was applied. Vacuum pressure was applied and resin was infused into the fiber. Once the resin was infused thoroughly and fiber was soaked with resin, the inlet line was shut and the entire set-up was placed in the oven for two hours at 175 °C to cure. The manufactured composite plate was also post cured at 80 °C for 12 h. Figure 3 shows a schematic of the setup for the VARTM process. The final composite plate had a thickness of 3 mm. Specimens for testing were cut with an Allied diamond saw to the dimensions of 3 × 12 × 60 mm.

### 2.3. Composite Characterization

Frequency sweeps were performed using a Dynamic Mechanical Analyzer Q800 by TA Instruments, New Castle, DE, USA. A dual cantilever fixture was used to perform tests in temperature step/strain mode with the strain amplitude of 0.1%. The frequency range was 0.1–10 Hz in log mode with five measurements in each decade. Storage modulus, loss modulus and tan δ values were recorded at temperatures 30, 40, 50, 60 and 70 °C.

Both short-term and long-term creep tests were also performed with the same equipment using a dual cantilever fixture. For short-term creep tests DMA was used in the creep TTS mode at the same temperatures as frequency sweep tests. Specimens were soaked for 8 min at each temperature and then under constant stress of 4.5 MPa for 12 min. A long-term creep test was also performed using a dual cantilever fixture and in the DMA creep mode. One creep test was performed at 30 °C with the constant stress of 4.5 MPa for 24 h and strain data were collected.

## 3. Results and Discussion

### 3.1. Resin Properties

The synthesis of MESS (Figure 2) was catalyzed using AMC-2, which also suppresses the hydroxyl-epoxy side reactions. The inhibitor, hydroquinone, was used in order to prevent premature polymerization. The final resin was fully characterized via acid number titration, ^1^H NMR, FTIR, GPC, and rheometry. The results are summarized in Table 1.

**Figure 4:**
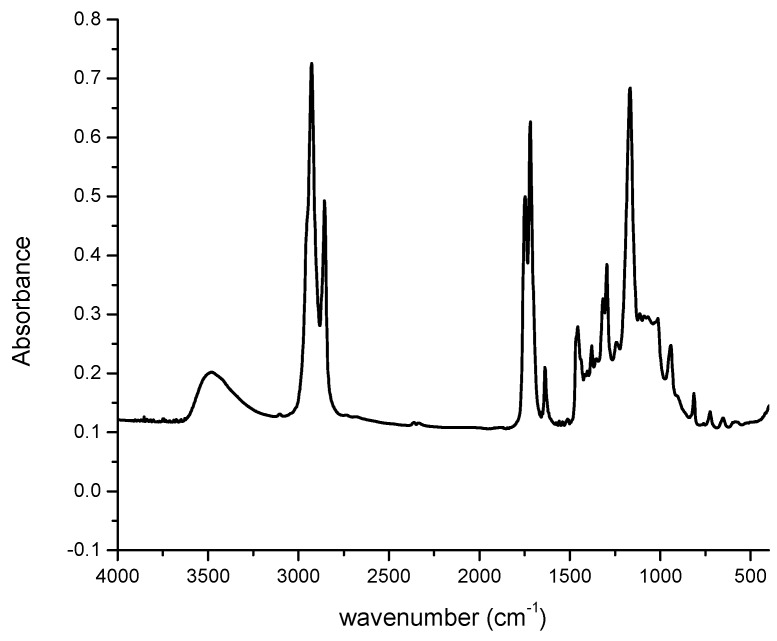
Fourier transform infrared spectrum of MESS.

The structure of the resin was characterized via FTIR and it is shown in Figure 4. The band around 3480 cm^−1^ was characteristic absorption of the O–H stretching while 1747 cm^−1^ was that of the C=O stretching of the fatty acid chains. The bands around 1718, 1637, and 941 cm^−1^ were assigned to the absorptions of C=O stretching, C=C stretching, and =C–H out of plane bending of methacrylates, respectively. The characteristic band for the oxirane C–O is absent after methacrylation. The summarized assignments are presented in Table 2.

**Figure 5:**
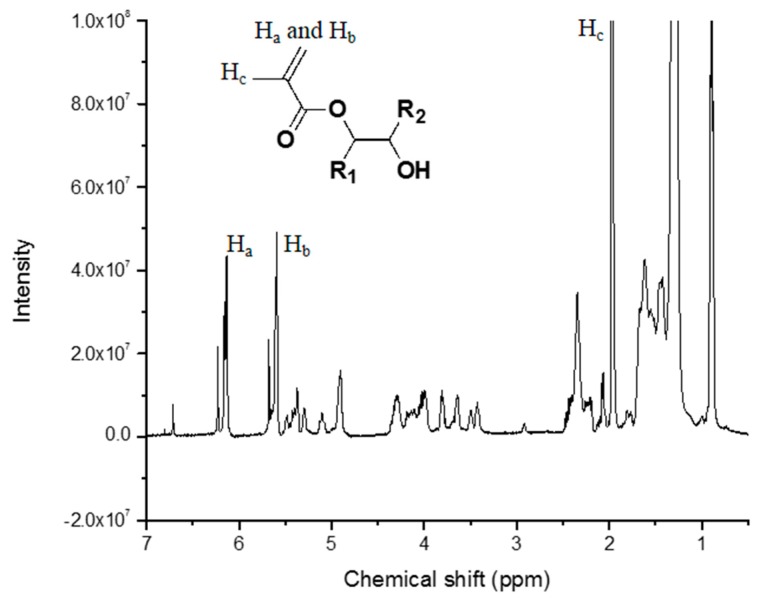
Proton nuclear magnetic resonance spectrum of the methacrylated epoxidized sucrose soyate (MESS) in CDCl_3_.

The structure of MESS was further characterized by ^1^H NMR and results are presented in Figure 5. The protons from the starting epoxy are typically between 2.8–3.2 ppm. As seen in Figure 5, those peaks are very minimal. Meanwhile, new peaks around 6.1(H_a_), 5.6(H_b_), and 1.9(H_c_) ppm appeared. These correspond to the protons in the vinyl and methyl groups of methacrylate. These results confirm the structure of MESS and indicate successful conversion of the oxiranes to methacrylates. Detailed analysis of the resin can be found in the published work of Yan and Webster [15].

### 3.2. Mechanical Properties of the Composite

Composite specimens were prepared, as explained in Section 2.2; the resulting fiber volume fraction of the manufactured composite was 35%.

**Figure 6:**
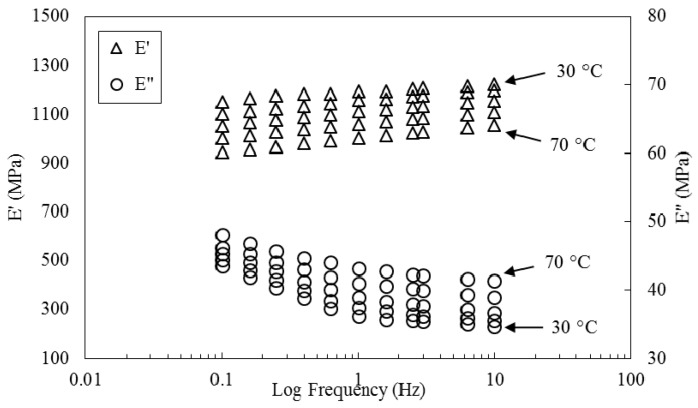
Frequency sweep of flax/MESS composite at different temperatures.

**Figure 7:**
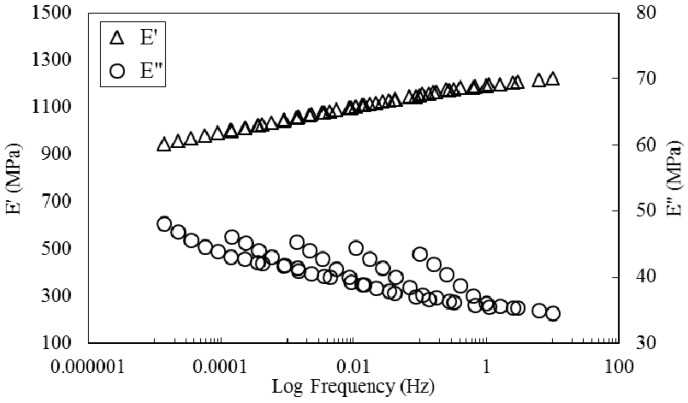
Master curves generated by solely horizontal shifting of storage modulus curve and using the same shift factors for loss modulus curves.

Results of frequency sweeps are presented in Figure 6. It is worth mentioning that, because *tan* δ is the ratio of loss modulus to storage modulus, it has not been plotted in the graphs. A horizontal shifting of the storage modulus values was performed using TA Instruments Data Analysis software. To perform the shifting process, the storage modulus curve at 30 °C was picked as the reference curve, and all other curves were shifted to the left. In this process, the loss modulus curves were also shifted with the same values of shift factors. The resulting plots are presented in Figure 7.

According to TTS assumptions, the same shift factors should be valid for all viscoelastic parameters [21,34]. Based on Figure 7, although a smooth master curve is obtained for the storage modulus, the curve for loss modulus is not satisfactory. This is indication of the fact that only one set of horizontal shift factors is not enough for all three sets of curves.

**Figure 8:**
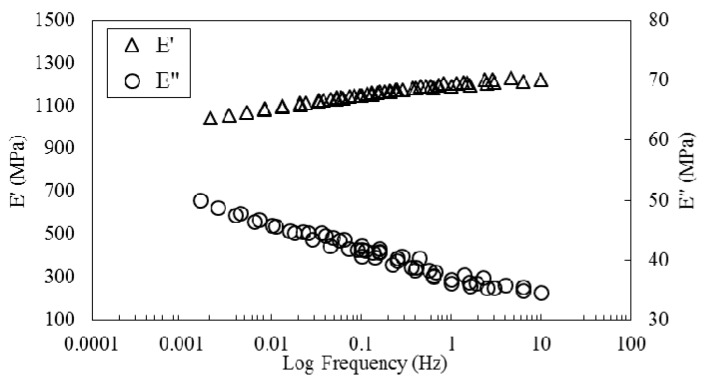
Master curves obtained by horizontal and vertical shifting of the frequency sweeps.

TA Instruments Data Analysis software was employed to move curves simultaneously and shift them both horizontally and vertically. A two-dimensional minimization method was used by the software and storage modulus, loss modulus and tanδ curves were simultaneously moved horizontally and vertically until they superpose. In addition to auto generated shift factors, an excel spreadsheet was used to modify the horizontal and vertical shift factors manually. The resulting master curves are presented in Figure 8. Much smoother master curves are obtained with this approach. Based on these results it is valid to conclude that MESS resin reinforced with flax fiber is a thermorheologically complex material and, to generate a smooth master curve, both horizontal and vertical shift factors are necessary [35].

**Figure 9:**
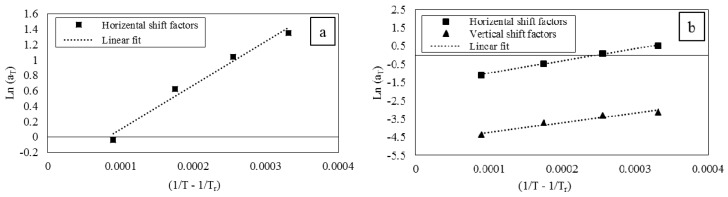
(**a**) Horizontal shift factors when only horizontal shift factors are used; (**b**) horizontal and vertical shift factors when both are used.

Horizontal shift factors used in Figure 7 are plotted in Figure 9a. Horizontal and vertical shift factors used in Figure 8 are plotted in Figure 9b. The dependency on temperatures of shift factors, both for horizontals and vertical shift factors, complies with the Arrhenius equation, which has the following form [1]:

(6) $$\ln\left(a_{T}\right)=\frac{Q}{R}\left(\frac{1}{T}-\frac{1}{T_{r}}\right)$$

where $a_{T}$ is the shift factor, *T_r_* (K) is the reference temperature and *T* (K) is an arbitrary temperature at which horizontal shift factor $a_{T}$ is desired. In this equation, *Q* is the activation energy (kJ/mol) and *R* is the universal gas constant (J/mol·K).

Based on Figure 9, the corresponding values for *Q* could be calculated using Equation (6). The calculated value for activation energy is 47.52 (kJ/mol) considering only horizontal shift factors of Figure 9a. If both horizontal and vertical shift factors of Figure 9b are considered, the values of *Q* are calculated to be 55.48 (kJ/mol) and 42.95 (kJ/mol) based on horizontal shift factors and vertical shift factors, respectively.

**Figure 10:**
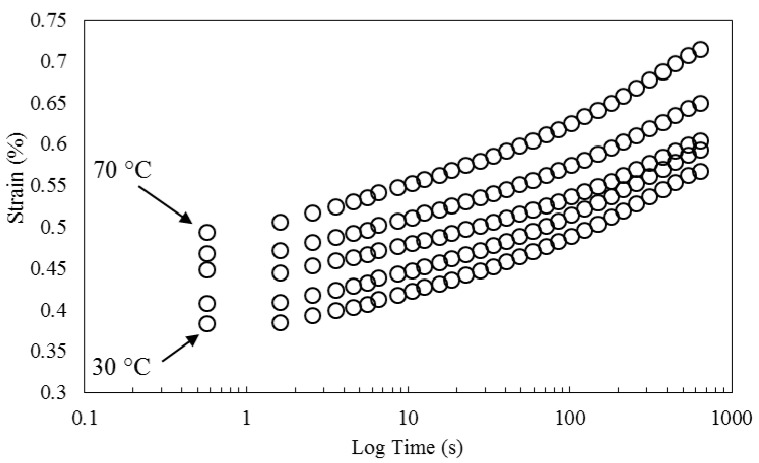
Creep strain *vs.* time at different temperatures.

**Figure 11:**
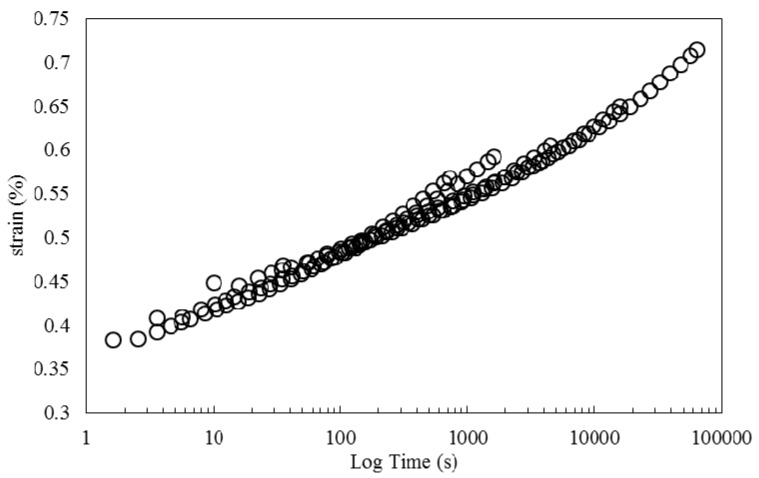
Creep strain master curve at 30 °C obtained by horizontal shifting of creep data at different temperatures.

Figure 10 shows creep data collected at different temperatures. Similar to the work of other researchers for natural fiber/thermoplastics [36] and natural fiber/thermosets [1], who have only applied horizontal shift factors, and neglected the application of shift factors to other viscoelastic properties, strain curves are horizontally shifted in reference to the strain curve at 30 °C to generate the creep master curves presented in Figure 11. As observed, an acceptably smooth master curve is obtained by this method.

**Figure 12:**
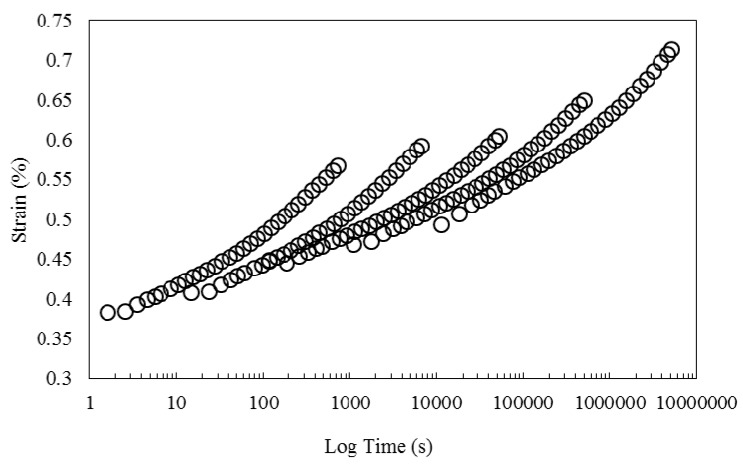
Creep strain curves at different temperatures shifted by the horizontal shift factors obtained from storage modulus master curve.

**Figure 13:**
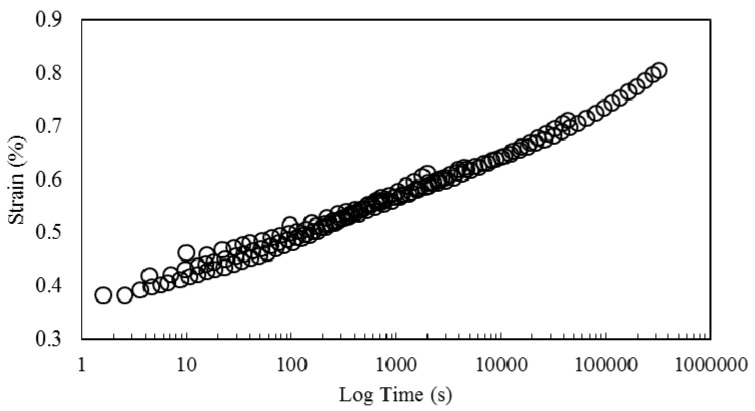
Creep strain master curve generated by horizontal and vertical shift factors.

Horizontal shift factors obtained from shifting the storage modulus curve in Figure 7 were applied. The results are presented in Figure 12. As can be seen, the creep curves do not superimpose and no satisfactory master curve is generated. Once more, this is an indication that horizontal shift factors are not solely sufficient to generate a master curve. In the next step, both horizontal and vertical shift factors obtained from shifting storage and loss modulus in Figure 8 is used to shift creep data. The result is a master curve shown in Figure 13. A smooth master curve is obtained by this method. In addition, by comparing the master curve obtained by horizontal shifting of creep data, with the curve obtained with horizontal and vertical shifts, it is perceived that the latter covers a wider range on the time axis.

**Figure 14:**
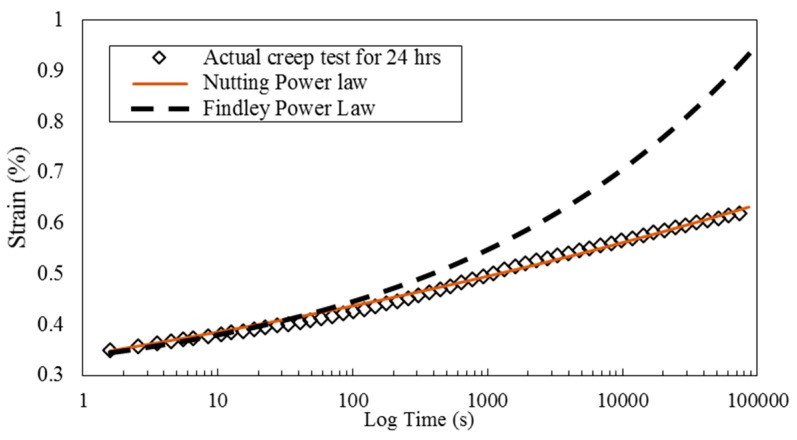
Comparison of extrapolated creep data with Nutting and Findley Power Laws with actual creep data for 24 h.

As mentioned before, a long-term creep test was performed at 30 °C for 24 h to check the validity of the obtained master curves. Creep data at 30 °C were used to find the parameters in Findley Power Law and Nutting Power Law. The parameters then were used to extrapolate the creep data to 24 h. Extrapolated curves based on Findley Power Law and actual creep data for 24 h are presented in Figure 14. At longer times, there is deviation between Findley model and actual creep data. However this model over estimates the strain creep values therefore provides more conservative values of creep strain. On the other hand, Nutting Power Law has a much better estimate of the creep data and stays closer to actual data.

**Figure 15:**
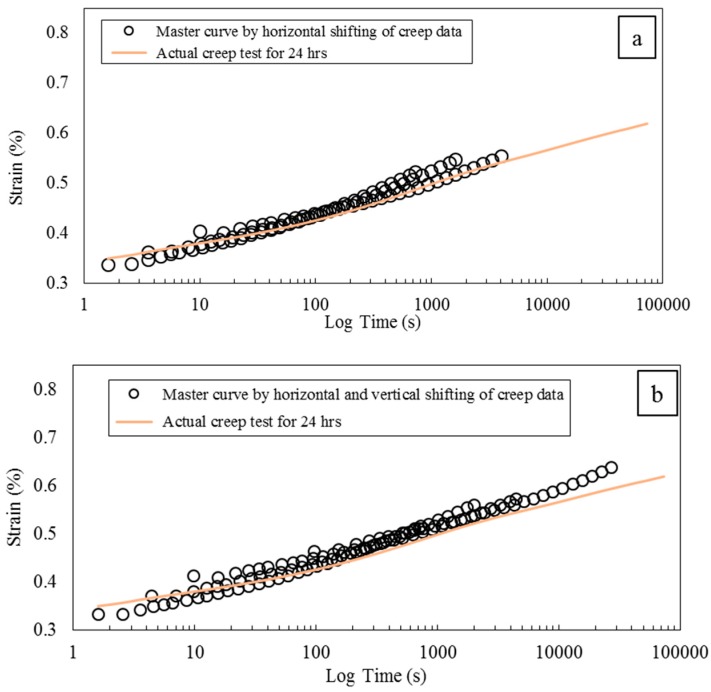
Comparison of actual creep data for 24 h with (**a**) master curve generated by horizontal shifting of creep data; (**b**) master curve generated by horizontal and vertical shift of creep data.

Figure 15 shows the comparison of the actual creep data with two master curves generated with horizontal shift factors, and horizontal and vertical shift factors. In both curves, there is a deviation from the actual creep data at longer times and both master curves tend to underestimate the creep strain.

Behera *et al.* [37] reinforced soy milk based composites with jute to investigate the properties of these composites and the viability of their use in packaging, furniture and automotive industries. They studied non-woven and woven jute soy composites using series of mechanical testing and characterized, strength, flexibility, hydrophilicity, surface characteristics, and susceptibility to biological degradation. Results of their study showed that developed composites are a feasible option for housing and office space applications as well as automotive industry. In another study, O’Donnell *et al.* [38] used vacuum assisted resin transfer molding (VARTM) to reinforce a soy-oil based resin (acrylated epoxidized soybean oil) with flax fiber with different fiber volume fractions, and studied the mechanical and thermal properties of the composites. They found that 33.3% styrene content in the resin was the optimum ratio of styrene to provide the maximum composite properties while maintaining the minimum styrene content possible. They run Dynamic Mechanical Analysis on their samples and measured loss and storage moduli of their composites. Similar to Behera *et al.* [37], their findings revealed that manufactured composites are suitable for applications in housing and automotive industries.

There have been other studies on developing vegetable oil-based or soy-based resins to be used in composites [39,40,41,42,43]. Most of these studies have focused on the synthesis or curing mechanism of bio-based resins; however, to the best of the authors’ knowledge, there have not been any studies on creep behavior or application of TTS methods to flax fiber reinforced soy-based thermosets. The authors believe that the findings and results of current study will be a great contribution in this area and valuable for further developments of their application in more engineering and structural applications.

## 4. Conclusions

In order to study the thermal and mechanical behavior of methacrylated epoxidized sucrose soyate (MESS) resin reinforced with flax fiber, frequency scans of flax/MESS composites were obtained at different temperatures and storage modulus, loss modulus (and tan δ as the ratio of these two functions) were recorded. The application of horizontal and vertical shift factors to loss modulus and storage modulus were studied. In addition, short-term strain creep at different temperatures were measured and curves were shifted with solely horizontal, and with both horizontal and vertical shift factors. The resulting master curves were compared with a 24-h creep test and two extrapolated creep models that were mentioned previously. It was shown that the use of solely horizontal shift factors are not adequate to achieve a smooth creep master curve for all viscoelastic properties of these composites. In addition, the use of both horizontal and vertical shift factors will result in a broader range of time.

Comparing the Findley and Nutting models with the actual creep data for 24 h, it was observed that the former overestimated the creep strain at longer times and deviated from the actual data, while the latter showed good agreement with the experimental data. The deviation of Findley power law from experimental data could be a sign that the parameters in mentioned model are more dependent on temperature and therefore the effect of temperature is more pronounced compared to time.

## Figures and Tables

**Table 1:** Properties of MESS.

	Acid number	% Solid	Viscosity (Pa·s) ^a^	*M*_n_ (kg/mol) ^b^	*Đ* ^c^
MESS	19	98.51	438.73	3,610	1.009

^a^ Measured by rheometry at 25 °C, taken at a frequency of 10 MHz; ^b^ Measured by GPC; ^c^ Polydispersity index.

**Table 2:** Functional group assignments on the MESS FTIR spectrum.

Wavenumber (cm^−1^)	Vibration type
3,480	OH stretching
1,747	Fatty acid ester C=O stretching
1,718	Methacrylate C=O stretching
1,637	Methacrylate C=C stretching
941	Methacrylate =C–H out of plane bending
